# Arabin Cerclage Pessary as a Treatment of an Acute Urinary Retention in a Pregnant Woman with Uterine Prolapse

**DOI:** 10.1155/2013/161376

**Published:** 2013-06-27

**Authors:** Alicia Martínez-Varea, Francisco Nohales-Alfonso, Vicente José Diago Almela, Alfredo Perales-Marín

**Affiliations:** Department of Obstetrics and Gynecology, Hospital Universitario y Politécnico La Fe, Torre F Planta 3*ª*, Bulevar Sur s/n, 46026 Valencia, Spain

## Abstract

A 35-year-old gravida 7, para 1, and abortus 5 female with hypogastric pain and inability to void urine after 14 + 3 weeks of amenorrhea was examined in the emergency department. One year before, a uterine prolapse had been diagnosed in another hospital. Examination showed a uterine prolapse grade 2 with palpable bladder. The patient was unable to void urine. After a manual reduction of the uterine prolapse, the patient underwent an emergency catheterization for bladder drainage. A Hodge pessary (size 70) was placed, which led to spontaneous micturitions. Due to the persistence of the symptoms the following day, Hodge pessary was replaced by an Arabin cerclage pessary. Although the pessary could be removed from the beginning of the second trimester, due to the uterine prolapse as a predisposing factor in the patient and the uncomplicated progression of pregnancy, it was decided to maintain it in our patient. Therefore, Arabin cerclage pessary allowed a successful pregnancy outcome and was not associated with threatened preterm delivery or vaginal infection.

## 1. Introduction

Acute urinary retention (AUR) is defined as the inability to void urine, with a retained volume of urine of 200 mL or greater [1]. AUR in early pregnancy is a very rare complication which leads to a real emergency [2].

## 2. Case Presentation

A 35-year-old gravida 7, para 1, and abortus 5 female with hypogastric pain and inability to void urine after 14 + 3 weeks of amenorrhea was examined in the emergency department. One year before, a uterine prolapse had been diagnosed in another hospital.

**Figure 1:**
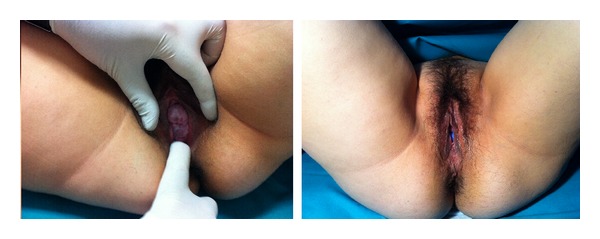
Arabin cerclage pessary as a treatment for uterine prolapse in a pregnant woman.

Examination showed an anteverted uterus and uterine prolapse grade 2 with palpable bladder. The patient was unable to void urine. Ultrasound revealed a cervical length of 30 mm and a singleton, cephalic fetus. A manual reduction of the uterine prolapse was made, and the patient underwent an emergency catheterization for bladder drainage (500 mL urine). No urinary tract infection was found. Then, it a Hodge pessary (size 70), and was placed after a spontaneous micturition, the woman was discharged for further ambulatory followup. The following day, the patient was admitted in to the emergency department because of a new AUR. Hodge pessary was replaced by another one of size 75, and due to the persistence of the symptoms, finally an Arabin cerclage pessary was placed (Figure 1). The patient was discharged after a rapid resolution of symptoms. 

Obstetric controls, which included vaginal cultures, were made every two weeks during the whole pregnancy. With negative vaginal cultures, the pregnancy progressed without incidences. The woman was admitted into the hospital at 36 + 5 weeks of gestation, due to a premature rupture of membranes. No evidence of vaginal infection was found. Then, Arabin cerclage pessary was removed, and the patient underwent a vaginal delivery, giving birth to a boy weighing 2,650 g. 

## 3. Discussion

AUR has been described in all trimesters but is commonly seen between the 10th and 16th weeks of gestation when the enlarging, retroverted, and gravid uterus becomes impacted within the pelvis and causes extrinsic compression of the urethra [3–5]. Urinary retention in pregnancy is an emergency and a failure to make a prompt diagnosis, and institutional treatment rapidly will result in irreversible uterine ischemia and spontaneous abortion, rupture of the uterus or bladder, rectal gangrene, intrauterine infection, or death [3].

AUR during pregnancy may appear due to a retroflexed uterus, lumbar disc herniation, paraurethral abscess, breech presentation, ectopic pregnancy, and conversion psychological disorder [6]. 

The first action to take after a rapid physical examination of a pregnant woman with AUR is the drainage of the bladder by catheterization and manual reduction of the uterine prolapse. A pessary can then be placed to keep the uterus in an anterior position and maintain a normal vesicourethral angle [2]. 

Although the pessary can be removed since the beginning of the second trimester [2], due to the uterine prolapse as a predisposing factor in the patient and the uncomplicated progression of pregnancy, it was decided to maintain it. 

To our knowledge, this is the first case report in which the efficacy of Arabin pessary has been showed, after the ineffectiveness of Hodge pessary, in the management of AUR in a pregnant woman with uterine prolapse. The use of Arabin pessary allows a successful pregnancy outcome, and it is not associated with threatened preterm delivery or vaginal infection.
